# Factors predicting long-term comorbidities in patients with Cushing’s syndrome in remission

**DOI:** 10.1007/s12020-018-1819-6

**Published:** 2018-11-22

**Authors:** Marie Helene Schernthaner-Reiter, Christina Siess, Alois Gessl, Christian Scheuba, Stefan Wolfsberger, Philipp Riss, Engelbert Knosp, Anton Luger, Greisa Vila

**Affiliations:** 1 Clinical Division of Endocrinology and Metabolism, Department of Internal Medicine III, Medical University of Vienna, Vienna, Austria; 2 Department of Surgery, Medical University of Vienna, Vienna, Austria; 3 Department of Neurosurgery, Medical University of Vienna, Vienna, Austria

**Keywords:** Cushing’s disease, Hypercortisolism, Remission, Diabetes, Hypertension, Obesity

## Abstract

**Purpose:**

In Cushing’s syndrome, comorbidities often persist after remission of glucocorticoid excess. Here, we aim to identify factors predicting long-term comorbidities in patients with Cushing’s syndrome in remission.

**Methods:**

In a retrospective cross-sectional study, 118 patients with Cushing’s syndrome in remission (52 pituitary, 58 adrenal, 8 ectopic) were followed for a median of 7.9 years (range 2–38) after the last surgery. Associations between baseline anthropometric, metabolic, hormonal parameters at diagnosis, and comorbidities (obesity, diabetes, hyperlipidemia, hypertension, osteoporosis, depression) at last follow-up, were tested by uni- and multivariate regression analysis.

**Results:**

In patients with manifest comorbidities at diagnosis, remission of Cushing’s syndrome resolved diabetes in 56% of cases, hypertension in 36% of cases, hyperlipidaemia in 23%, and depression in 52% of cases. In a multivariate regression analysis, age, fasting glucose, BMI, and the number of comorbidities at diagnosis were positive predictors of the number of long-term comorbidities, while baseline 24-h urinary free cortisol (UFC) negatively correlated with the persistence of long-term comorbidities. The negative relationship between baseline UFC and long-term comorbidities was also found when pituitary and adrenal Cushing’s cases were analyzed separately. Baseline UFC was negatively related to the time of exposure to excess glucocorticoids.

**Conclusions:**

Long-term comorbidities after remission of Cushing’s syndrome depend not only on the presence of classic cardiovascular risk factors (age, hyperglycemia, BMI), but also on the extent of glucocorticoid excess. Lower baseline UFC is associated with a higher number of long-term comorbidities, possibly due to the longer exposure to excess glucocorticoids in milder Cushing’s syndrome.

## Introduction

Glucocorticoid excess in Cushing’s syndrome leads to complications such as increased visceral adipose tissue, obesity, impaired glucose tolerance, diabetes mellitus, hypertension, hyperlipidemia, osteoporosis, and neuropsychiatric comorbidities, and is associated with a significantly increased mortality [1–4]. The fact that many of these diseases are quite prevalent in the general population often leads to difficulties and delays in the diagnosis of Cushing’s syndrome.

Among all clinical features accompanying Cushing’s syndrome, weight gain is the most prevalent one and reported in up to 82% of cases, with overweight described in 21–48% and obesity in 32–41% of patients [5–8]. The most prevalent comorbidity is hypertension, which is reported in 50–85% of patients with Cushing’s syndrome, often being also the presenting comorbidity [5–7, 9, 10]. Not only the accompanying metabolic syndrome, but also upregulation of the sympathetic nervous system and water retention caused by the mineralocorticoid properties of glucocorticoids play a role in the pathophysiology of glucocorticoid-induced hypertension [11]. Glucocorticoids impair both insulin secretion and insulin sensitivity, and over time contribute to the failure of beta-cells to adequately enhance insulin secretion compensating for the increased insulin resistance, and therefore to the development of diabetes [12]. Impaired glucose tolerance is reported in 14–64% of patients with Cushing’s syndrome, diabetes mellitus in 13–47% [5–8, 10]. Additional prevalent comorbidities include hyperlipidemia, osteoporosis, and depression/psychopathology [6].

The severity of Cushing’s syndrome does not correlate with the extent of hypercortisolism [13]. Instead, diabetes and hypertension were found to be associated with older age and a longer exposure to excess glucocorticoids [7, 8]. Older age and time of exposure to excess glucocorticoids were also found to predict mortality in patients with Cushing’s disease [3].

The primary goal in the therapy of Cushing’s syndrome is the cure of hypercortisolism, and the therapy of choice is surgery [2, 14]. Successful surgery and remission of glucocorticoid excess often lead to amelioration of accompanying signs and symptoms, but comorbidities are not always reversible [1, 6, 15]. As most Cushing’s comorbidities (abdominal obesity, hypertension, insulin resistance, diabetes mellitus, and hyperlipidemia) are also classical cardiovascular risk factors, the persistence of comorbidities confers an increased risk for cardiovascular mortality, which remains increased also decades after Cushing’s remission [4]. To date, there are no data on factors associated with the long-term persistence of comorbidities in patients with Cushing’s syndrome.

In the present study, we aimed to investigate which baseline factors, present at the initial diagnosis of Cushing’s syndrome, predict the presence of long-term comorbidities. To address this question, we investigated a single-center cohort of patients with Cushing’s syndrome in long-term remission, evaluating the presence of comorbidities at the last visit and their relation to baseline parameters.

## Patients and methods

### Patient population

The study was approved by the Ethics committee of the University of Vienna (EK Nr. 1457/2016) and included patients with Cushing’s syndrome in remission that attended the endocrine outpatient clinic of the Medical University of Vienna between 2012 and 2018. Inclusion criteria were: (1) patients with biochemically diagnosed Cushing’s syndrome (pituitary, adrenal, or ectopic Cushing’s syndrome) who had undergone at least one surgery for removing the cause of hypercortisolism in our hospital, (2) disease remission since at least 2 years after the last surgery, and (3) no requirement for medical treatment of hypercortisolism after the last surgery. Exclusion criteria were: (1) active Cushing’s syndrome and/or concomitant medical therapy for the treatment of hypercortisolism, (2) histopathological evidence of adrenal carcinoma, and (3) concomitant malignant disease, which was not in complete remission at the time of last follow-up.

### Data collection

Data on clinical and biochemical parameters, comorbidities at diagnosis of Cushing’s syndrome and at the last follow-up visit, treatment modalities, surgical protocols and histology results were retrospectively extracted from the medical records by two researchers independently (first researcher performing the initial data extraction from medical records, second researcher double-checking the data comparing the extracted information with hospital records).

### Baseline visit

The diagnosis of Cushing’s syndrome was performed in our center based on the presence of at least three of the following parameters: cortisol after 1 mg overnight dexamethasone test, cortisol after Liddle’s test, 24-h urinary free cortisol (UFC), midnight cortisol, cortisol and adrenocorticotropic hormone (ACTH) at fasting and during corticotropin-releasing hormone (CRH)-test. The following data from baseline work-up were retrospectively extracted from the medical records: serum cortisol, plasma ACTH, cortisol after 1 mg overnight dexamethasone test, cortisol after Liddle’s test, UFC, weight, height, blood pressure, fasting glucose, glycated hemoglobin (HbA1c), plasma glucose 120 min after a 75 g oral glucose tolerance test (OGTT, where available), fasting triglycerides, fasting cholesterol, high-density lipoprotein (HDL)-cholesterol, low-density lipoprotein (LDL)-cholesterol, creatinine, estimated glomerular filtration rate (GFR), pituitary hormone levels, current medication, current comorbidities and the time of disease manifestation (time of appearance of first symptoms or comorbidities). Diagnostic delay was calculated as the time from appearance of first symptoms/comorbidities to diagnosis. As UFC was determined on two separate occasions in most patients, the mean of both measurements was calculated and included in further analysis.

### Follow-up visits

During routine outpatient visits, remission of Cushing’s syndrome was assessed by clinical examination, fasting serum cortisol, plasma ACTH, 1 mg dexamethasone suppression test and 24-h UFC. In patients who were receiving glucocorticoid replacement, only baseline serum cortisol and plasma ACTH were determined. Information on all previous treatment modalities for Cushing’s syndrome was extracted from hospital records. In addition, the following data were obtained at the most recent follow-up visit: weight, height, blood pressure, fasting glucose, HbA1c, glucose 120 min after an OGTT (where available), fasting triglycerides, fasting cholesterol, HDL-cholesterol, LDL-cholesterol, creatinine, GFR, pituitary hormone levels, current medication and current comorbidities. Exposure time to excess glucocorticoids was calculated as the time from first disease manifestation to biochemical remission.

### Evaluation of comorbidities

Hypertension was diagnosed if systolic blood pressure was above 140 mm Hg and/or diastolic blood pressure was above 90 mm Hg and/or patients were taking antihypertensive medication [16]. Body mass index (BMI) was calculated as weight in kilograms divided by the square of height in meters. Overweight was diagnosed in patients with BMI ≥ 25 and < 30 kg/m^2^, obesity was diagnosed in patients with BMI 30 kg/m^2^ or higher. Diabetes mellitus was diagnosed when fasting plasma glucose was ≥ 126 mg/dL, and/or HbA1c ≥ 6.5%, and/or random plasma glucose or glucose 2 h after an OGTT were ≥ 200 mg/dL or when patients were taking antidiabetic medication [17]. In patients without diabetes, impaired fasting glucose was diagnosed when fasting plasma glucose levels were between 100 and 125 mg/dL, impaired glucose tolerance was diagnosed when 2 h-glucose in an OGTT was between 140 and 199 mg/dL or when HbA1c was between 5.7 and 6.4%; the presence of either impaired fasting glucose or impaired glucose tolerance was summarized as prediabetes [17]. Hyperlipidemia was diagnosed when either total cholesterol was ≥ 200 mg/dL or triglycerides were ≥ 150 mg/dL or when patients were taking lipid-lowering medication, whereas hypertriglyceridemia was diagnosed when triglycerides were ≥ 150 mg/dL [18]. The metabolic syndrome was defined according to the WHO criteria [19]. All patients with depressive symptoms were referred to the psychiatrist; patients were considered to suffer from depression if they were taking antidepressive medication, or if the initiation of antidepressive medication was recommended. Osteoporosis/osteopenia were diagnosed by dual-energy X-ray absorptiometry examinations at the lumbar spine and hip, which were mainly performed outside our medical center. Osteopenia was diagnosed when the T-score was between –1 and –2.5 SD and osteoporosis when the T-score was under –2.5 SD. All diagnosed comorbidities were treated, respectively. In the present study, we report comorbidities present at two time-points: (1) diagnosis of Cushing’s syndrome and (2) most recent follow-up visit.

### Assays

All biochemical parameters were determined in the routine certified laboratory of our university hospital (www.kimcl.at). UFC was determined after extraction (liquid/liquid with dichloromethane) by electrochemiluminescence immunoassay (ECLIA, Modular Elecsys E170, Roche) with an intra-assay coefficients of variation (CV) of < 5%. Cortisol was measured using ECLIA, with sensitivity and CV being 0.04 g/dL and 6%, respectively.

### Statistical analysis

Data was tested for normality using Shapiro–Wilk test. Parametric data are represented as mean ± standard error of the mean (SEM) and nonparametric data are given as median ± interquartile range (IQR) unless stated otherwise. Differences between means were tested by Student’s *t*-tests, Mann–Whitney-*U*-tests, Kruskal–Wallis tests or analysis of variance (ANOVA) as stated. Univariate correlations were computed by Spearman’s rank correlation coefficient. Stepwise multiple regression was performed with selected parameters correlating significantly in univariate analysis. Logistic regression analysis was performed with selected comorbidities (hypertension, diabetes mellitus, obesity, and hyperlipidemia) as outcome measures and baseline parameters (age, BMI, glucose, triglycerides, and UFC at Cushing’s diagnosis) as independent variables. Distribution of frequencies was compared by Pearson’s Chi-squared test. Data were analyzed using the SPSS statistics software (IBM).

## Results

### Baseline characteristics and differences between pituitary and adrenal Cushing’s syndrome

**Fig. 1:**
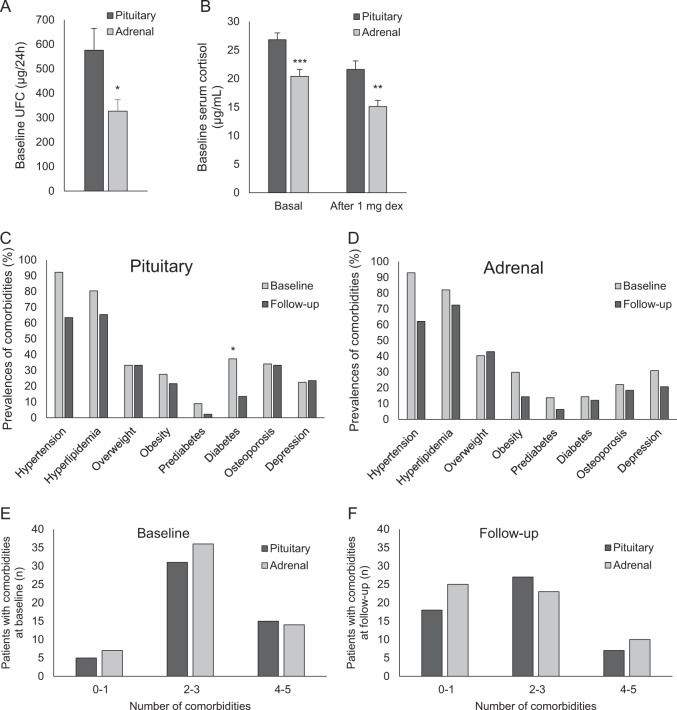
Differences between pituitary and adrenal Cushing’s syndrome at diagnosis (baseline) and last follow-up. Twenty-four hour urinary free cortisol (UFC) at diagnosis **a** and basal and 1 mg dexamethasone (dex) suppressed morning serum cortisol at diagnosis **b**. Prevalence of comorbidities (hypertension, hyperlipidemia, overweight, obesity, prediabetes, diabetes, osteoporosis and depression) at baseline and follow-up in percent (of cases with known status) in pituitary **c** and adrenal Cushing’s syndrome **d**. Frequency of patients according to number of comorbidities (including hypertension, hyperlipidemia, obesity, diabetes mellitus, osteoporosis and depression) at time of diagnosis **e** and at last follow-up **f**. Significances indicate comparisons between pituitary and adrenal Cushing’s syndrome. Differences between groups were tested by independent samples Mann–Whitney-*U-*tests (a and b) or Pearson’s Chi-squared test (c and d). n.s. not significant, *p < 0.05, **p < 0.005, ***p < 0.001

Our cohort consisted of 118 patients with Cushing’s syndrome in remission: 52 with Cushing’s disease, 58 with adrenal Cushing’s syndrome (adrenal adenoma *n* = 49, bilateral adrenal hyperplasia *n* = 6, two unilateral adrenal adenomas *n* = 1, unilateral adrenal hyperplasia *n* = 1; histopathology was not available for 1 case), and 8 with ectopic Cushing’s syndrome. Baseline characteristics of patients of all groups are given in Table 1. Patients with Cushing’s disease were significantly younger at diagnosis compared to those with adrenal Cushing’s syndrome. The exposure time to excess glucocorticoids (time from initial manifestation to biochemical remission) was higher in patients with Cushing’s disease compared to those with adrenal and ectopic Cushing’s syndrome (Table 1). Patients with Cushing’s disease had a higher degree of hypercortisolism when compared to adrenal Cushing’s syndrome (Fig. 1a, b). Blood pressure and LDL-cholesterol were lower in Cushing’s disease when compared to adrenal Cushing’s syndrome (*p* = 0.031 and *p* = 0.046, respectively). There were no differences in other biochemical parameters between pituitary and adrenal subgroups.

**Table 1:** Baseline characteristics at diagnosis of Cushing’s syndrome and treatment modalities

	All patients	Pituitary	Adrenal	Ectopic	*p*
*n*	118	52	58	8	
Gender m/f *n* (%)	24/94 (20.3/79.7)	14/38 (26.9/73.1)	8/50 (13.8/86.2)	2/6 (25.0/75.0)	n.s.
Age at diagnosis (years)	42.8 (1.3)	38.7 (1.7)^a^	46.4 (1.8)	43.4 (5.6)	0.012
Diagnostic delay (months)	25.8 (48.6)^b^	31.6 (45.5)^b^	16.5 (53.0)^b^	18.0 (4.7)	0.046
Exposure time to excess glucocorticoids (months)	30.5 (62.1)^b^	40.5 (83.3)^b,c^	26.3 (51.6)^b^	19.9 (5.0)	0.006
Age at last follow-up (years)	54.9 (1.3)	53.9 (2.0)	55.9 (1.9)	53.4 (6.1)	n.s.
Time since last surgery (years)	7.9 (12.7)^b^	12.7 (14.5)^b,d^	5.9 (8.7)^b^	9.0 (1.1)	0.027
Number of surgeries	1 (0)	1 (1)^e^	1 (0)	1 (0)	0.007
Bilateral ADX *n* (%)	28 (23.7)	13 (25.0)	7 (12.1)^f^	8 (100.0)	< 0.001

Data shown are means ± SEM for parametric data or medians ± IQR for nonparametric data. *p* indicates the level of significance for the overall difference between all three groups, tested with one-way ANOVA or independent samples Kruskal–Wallis-tests or with Pearson’s Chi-Squared tests. Significant results between single groups, as evaluated by post-hoc testing or adjusted residuals are shown with footnotes a and c–e. Number of surgeries indicates surgeries for Cushing's syndrome until remission from hypercortisolism was achieved

*ADX* adrenalectomy

^a^*p* = 0.009 vs. adrenal, n.s. vs. ectopic

^b^Nonparametric

^c^*p* = 0.039 vs. adrenal, *p* = 0.031 vs. ectopic

^d^*p* = 0.022 vs. adrenal, n.s. vs. ectopic

^e^Significant vs. adrenal, n.s. vs. ectopic

^f^Significant vs. ectopic, n.s. vs. pituitary

### Baseline comorbidities

The prevalences of comorbidities in pituitary and adrenal Cushing’ syndrome are shown in Fig. 1c, d. Diabetes was 2.4-fold more frequent in Cushing’s disease when compared to adrenal Cushing’s syndrome (*p* = 0.006). Metabolic syndrome was present in 39.3% of all patients. The total number of comorbidities (which included hypertension, hyperlipidemia, obesity, diabetes mellitus, osteoporosis, and depression) in pituitary and adrenal Cushing’s are shown in Fig. 1 e, f. The number of comorbidities at baseline was significantly associated with BMI (*R* = 0.296, *p* = 0.001), fasting glucose (*R* = 0.441, *p* < 0.001), HbA1c (*R* = 0.419, *p* < 0.001) and triglycerides (*R* = 0.284, *p* = 0.003) but was not related to parameters of hypercortisolism, including UFC, baseline cortisol or cortisol after dexamethasone suppression test (data not shown).

We did not detect any significant differences in baseline number of comorbidities, age at diagnosis, BMI, fasting glucose, HbA1c, triglycerides, GFR or UFC when comparing patients diagnosed before 2005 (*n* = 56) with those diagnosed during or after 2005 (*n* = 62).

### Treatment modalities for Cushing’s syndrome

Remission after a single surgery was achieved in 98 patients (83.1%), and 28 patients (23.7%) received bilateral adrenalectomy. Patients with Cushing’s disease required significantly more surgeries before achieving biochemical remission (excluding debulking and Nelson’s syndrome surgeries) (Table 1). Bilateral adrenalectomy was performed in men more frequently (*p* = 0.014, men 10/24 vs. women 18/94).

### Long-term follow-up and comorbidities

After a follow-up of 7.9 years after remission (range 2–38 years), significant improvements in BMI, blood pressure, parameters of glucose and lipid metabolism as well as in the total number of comorbidities were observed, while kidney function remained unchanged (Table 2). The prevalence of metabolic syndrome declined to 18.4%. Patients with adrenal disease had a significantly shorter follow-up than patients with pituitary disease, but age at the last follow-up visit was similar between the groups (Table 1). The number of comorbidities decreased in all three groups; distribution of numbers of comorbidities at last follow-up for pituitary and adrenal Cushing’s syndrome is shown in Fig. 1c, d. Hypertension and hyperlipidemia remained the most frequent comorbidities also at last follow-up. Remission rates of comorbidities as well as rates of new manifestation of comorbidities during follow-up are shown in Supplemental Table 1. Gender-specific differences in Cushing's comorbidities were detected in BMI at follow-up, which was significantly higher in men (*p* = 0.042). The presence or absence of specific medication for hypertension, diabetes, or hyperlipidemia at follow-up was not associated with the number of comorbidities at follow-up.

**Table 2:** Subgroup baseline and follow-up characteristics in the whole cohort, as well as in pituitary, adrenal, and ectopic Cushing’s syndrome

	Baseline	Follow-up	*p*
All cases (n = 118)			
BMI (kg/m^2^)	26.4 (6.8)*	25.9 (5.8)*	<0.001
MAP (mm Hg)	111.1 (1.4)	100.0 (1.3)	<0.001
Fasting glucose (mg/dL)	91.0 (31.0)*	89.0 (15.0)*	0.041
HbA1c (%)	5.9 (1.2)*	5.5 (0.7)*	<0.001
Triglycerides (mg/dL)	138.0 (124.0)*	111.0 (72.0)*	<0.001
Total cholesterol (mg/dL)	230.9 (4.9)	206.0 (45.0)*	<0.001
HDL cholesterol (mg/dL)	56.0 (23.0)*	58.0 (28.3)*	n.s.
LDL cholesterol (mg/dL)	140.1 (4.6)	121.0 (48.4)*	0.002
GFR (mL/min/1.73 m^2^)	78.8 (21.0)*	79.1 (1.9)	n.s.
Number of comorbidities	3 (2)	2 (2)	<0.001
**Pituitary (*n*** = 52)			
BMI (kg/m^2^)	26.1 (7.1)*	25.7 (6.2)*	n.s.
MAP (mm Hg)	107.7 (2.1)	100.6 (1.8)	0.007
Fasting glucose (mg/dL)	91.0 (57.5)*	89.0 (14.0)*	n.s.
HbA1c (%)	6.2 (1.8)*	5.6 (0.63)*	<0.001
Triglycerides (mg/dL)	147.5 (138.8)*	110.0 (76.0)*	0.001
Cholesterol (mg/dL)	221.5 (6.6)	209.0 (47.0)*	n.s.
HDL cholesterol (mg/dL)	53.0 (22.0)*	58.0 (26.5)*	n.s.
LDL cholesterol (mg/dL)	129.0 (6.5)	121.0 (46.8)*	n.s.
GFR (mL/min/1.73 m^2^)	80.3 (27.7)*	81.9 (2.6)	n.s.
Number of comorbidities	3 (2)	2 (2)	<0.001
**Adrenal (*n*** = 58)			
BMI (kg/m^2^)	26.0 (6.9)*	25.8 (5.2)*	0.001
MAP (mm Hg)	113.9 (1.9)	100.7 (1.8)	<0.001
Fasting glucose (mg/dL)	89.0 (13.0)*	89.0 (15.5)*	n.s.
HbA1c (%)	5.8 (0.9)*	5.5 (0.7)*	0.013
Triglycerides (mg/dL)	135.0 (96.0)*	109.0 (72.5)*	0.009
Cholesterol (mg/dL)	240.0 (7.0)	204.7 (4.9)	<0.001
HDL cholesterol (mg/dL)	59.6 (2.4)	57.0 (30.5)*	n.s.
LDL cholesterol (mg/dL)	147.2 (5.9)	118.9 (4.6)	0.001
GFR (mL/min/1.73 m^2^)	77.1 (2.0)	77.7 (2.8)	n.s.
Number of comorbidities	3 (1.5)	2 (2)	<0.001
**Ectopic (*n*** = 8)			
BMI (kg/m^2^)	29.9 (0.86)	28.9 (1.0)	n.s.
MAP (mm Hg)	113.1 (5.7)	91.3 (3.7)	0.012
Fasting glucose (mg/dL)	170.4 (32.5)	85.0 (67.0)*	0.028
HbA1c (%)	6.1 (1.2)*	5.4 (0.5)*	0.028
Triglycerides (mg/dL)	247.5 (51.4)	159.8 (26.8)	n.s.
Cholesterol (mg/dL)	239.9 (27.0)	223.8 (14.2)	n.s.
HDL cholesterol (mg/dL)	53.8 (5.4)	56.6 (4.5)	n.s.
LDL cholesterol (mg/dL)	142.4 (22.6)	135.2 (12.8)	n.s.
GFR (mL/min/1.73 m^2^)	84.9 (6.6)	71.8 (8.1)	n.s.
Number of comorbidities	4 (2)	2 (1)	0.008

Data shown are means ± SEM for parametric data or medians ± IQR for nonparametric data. Differences between groups were tested with related samples Wilcoxon Signed Rank tests, with paired samples Student’s t-tests or with related samples Friedman’s two-way ANOVA by ranks

*BMI* body mass index, *GFR* glomerular filtration rate, *HbA1c* glycated hemoglobin, *HDL* high-density lipoprotein, *LDL* low-density lipoprotein, *MAP* mean arterial pressure

*Nonparametric

### Predictors of long-term comorbidities

To identify baseline parameters that predict long-term comorbidities in Cushing’s syndrome, we performed univariate and multivariate correlation analyses. In the whole cohort, the number of long-term comorbidities correlated significantly with the following baseline parameters: age, fasting glucose, HbA1c, BMI, and triglycerides but not with cholesterol or its fractions. Negative correlations were detected between the number of long-term comorbidities and UFC in the whole cohort, as well as in pituitary and adrenal subgroups (Table 3). We also found a negative correlation between long-term comorbidities and serum cortisol after Liddle’s test in patients with adrenal Cushing’s syndrome. Diagnostic delay or exposure time to excess glucocorticoids did not significantly relate to the number of long-term comorbidities. Selected parameters correlating in univariate analysis were included in stepwise multiple regression analysis, which revealed age, number of comorbidities at diagnosis, baseline fasting glucose and BMI as independent positive predictors of the number of long-term comorbidities, and UFC level at diagnosis as an independent negative predictor of the number of long-term comorbidities (Table 3).

**Table 3:** Predictors of the number of comorbidities at long-term follow-up

	All		Pituitary	Adrenal
Baseline parameter	Univariate	Multivariate	Univariate	Univariate
	*R*	Standardized beta	*R*	*R*
Age at diagnosis	0.438***	0.222*	0.374*	0.543***
Number of comorbidities	0.487***	0.333**	0.538***	0.574***
BMI	0.236*	0.181*	0.352*	n.s.
Fasting glucose	0.281*	0.263*	n.s.	0.489***
HbA1c	0.340**		0.458*	0.358*
Triglycerides	0.253*	n.s.	0.433**	n.s.
HDL cholesterol	n.s.		−0.353*	n.s.
GFR	−0.268*	n.s.	−0.456**	n.s.
Serum cortisol post Liddle test	n.s.		n.s.	−0.408**
UFC	−0.362***	−0.376**	−0.460**	−0.337*
Diagnostic delay	n.s.		n.s.	n.s.
Exposure time to excess glucocorticoids	n.s.		n.s.	n.s.

Nonparametric correlation coefficients (*R*) and multiple regression of number of comorbidities at last follow-up with parameters at baseline. Selected parameters significantly relating to the number of comorbidities in univariate correlation analysis were included in stepwise multiple regression. Empty spaces in multivariate analysis signify that those parameters were not included in this analysis. HDL cholesterol, serum cortisol post Liddle test, diagnostic delay and exposure time to excess glucocorticoids were not included in multivariate analysis because they did not correlate in univariate analysis. HbA1c was not included in multivariate analysis due to concerns of multicollinearity with fasting glucose

*HbA1c* glycated hemoglobin, *HDL* high-density lipoprotein, *UFC* urinary free cortisol

*n.s.* not significant, *p < 0.05, **p < 0.005, ***p < 0.001

**Fig. 2:**
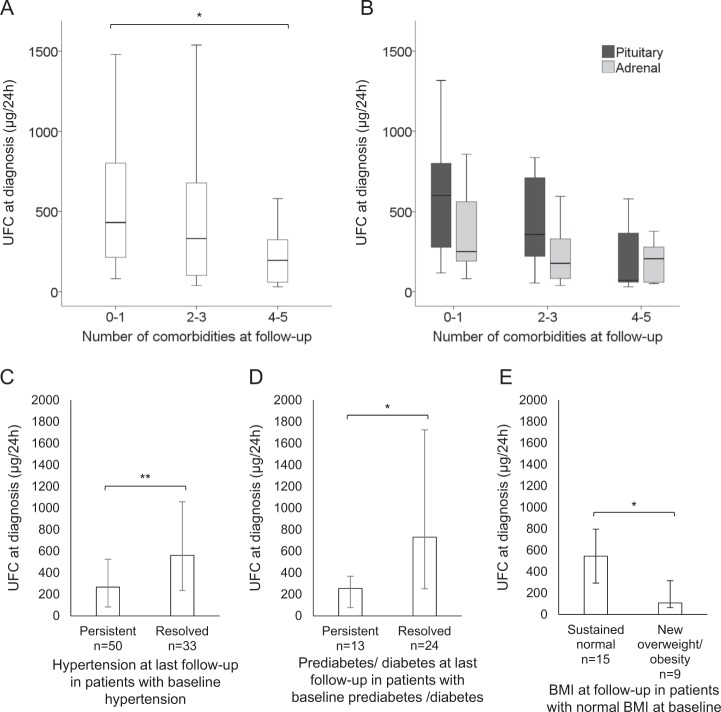
Levels of baseline 24-hour urinary free cortisol (UFC) in relation to long-term comorbidities. **a**, **b** Levels of baseline UFC in relation to the number of long-term comorbidities (including hypertension, hyperlipidemia, obesity, diabetes mellitus, osteoporosis, and depression) at last follow-up in the whole cohort **a** and shown separately in pituitary and adrenal Cushing’s syndrome **b**. **c**–**e** Differences in UFC of patients with comorbidities that resolved or newly manifested during follow-up. **c** Differences in baseline UFC concentrations between patients with hypertension at baseline, whose hypertension persisted (present, *n* = 50) or resolved (absent, *n* = 33) at long-term follow-up. **d** Differences in baseline UFC between patients with prediabetes or diabetes at baseline, depending on the presence (persistent, *n* = 13) or absence of prediabetes/diabetes (resolved, *n* = 24) at long-term follow-up. **e** Differences in baseline UFC between patients with normal BMI at baseline, who sustained normal BMI (sustained normal, *n* = 15) or became overweight/obese (*n* = 9) at long-term follow-up. Differences between means were tested with independent samples Kruskal–Wallis test **a**, with two-way ANOVA **b** or with independent samples Mann–Whitney-*U-*tests **c**–**e**. *p < 0.05, **p<0.005

Patients who had 4 or 5 comorbidities at long-term follow-up had significantly lower UFC levels at diagnosis of Cushing’s syndrome when compared to patients who had 0 or 1 long-term comorbidities (Fig. 2a). We detected no significant interaction between the number of comorbidities and pituitary vs adrenal disease in terms of UFC by two-way ANOVA (Fig. 2b).

We also found an association between remission of specific comorbidities and baseline UFC; of all patients with hypertension at baseline, the ones with higher baseline UFC achieved remission from hypertension at follow-up (Fig. 2c); the same was found regarding remission from prediabetes or diabetes mellitus (Fig. 2d). In addition, of all patients with normal weight at baseline, those who sustained their normal weight at last follow-up had significantly higher baseline UFC (Fig. 2e).

To investigate which factors are related to UFC, we computed correlations between UFC values and different baseline and follow-up parameters. UFC was significantly negatively associated with diagnostic delay and with the exposure time to excess glucocorticoids; these correlations remain significant in patients with Cushing’s disease but not in those with adrenal Cushing’s syndrome (Table 4). No correlations were detected between UFC and baseline characteristics or comorbidities.

**Table 4:** Relationship between UFC and baseline and follow-up characteristics

	All		Pituitary		Adrenal	
	*R*	*p*	*R*	*p*	*R*	*p*
Age at diagnosis	−0.451	< 0.001	−0.325	0.050	−0.619	< 0.001
Number of comorbidities at follow-up	−0.362	< 0.001	−0.460	0.004	−0.337	0.024
BMI at follow-up		n.s.	−0.354	0.034		n.s.
MAP at follow-up	−0.244	0.024		n.s.	−0.471	0.013
HbA1c at follow-up	−0.414	0.001	−0.473	0.015		n.s.
Diagnostic delay	−0.232	0.027	−0.299	0.073		n.s.
Exposure time to excess glucocorticoids	−0.244	0.020	−0.378	0.021		n.s.

Nonparametric correlation coefficients (*R*) and levels of significance (*p*) of urinary free cortisol with different baseline and follow-up parameters in all patients as well as subgroups (pituitary and adrenal Cushing’s syndrome)

*HbA1c* glycated hemoglobin, *MAP* mean arterial pressure

In turn, exposure time to excess glucocorticoids was positively associated with the number of surgeries (*R* = 0.357, *p* < 0.001), while diagnostic delay and exposure time to excess glucocorticoids were significantly higher in patients with adrenal insufficiency at last follow-up (*p* = 0.012 and *p* = 0.015, respectively, excluding those patients who had received bilateral adrenalectomy).

### Determinants of specific long-term comorbidities

**Fig. 3:**
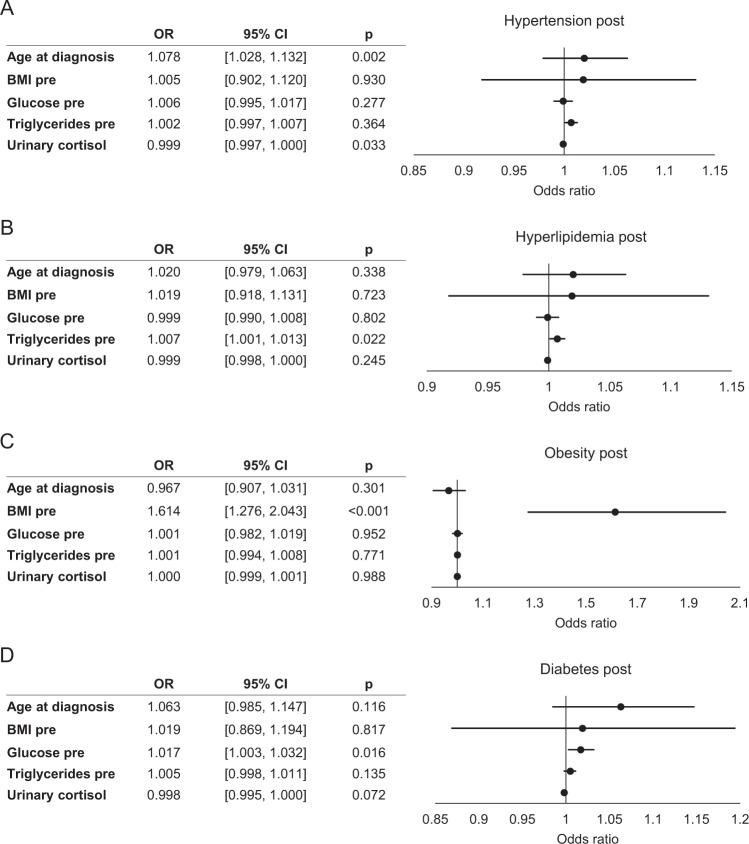
Logistic regression analyses of different comorbidities [Hypertension post **a**, Hyperlipidemia post **b**, Obesity post **c**, and Diabetes mellitus post **d**] at last follow-up (dependent variable) with predicting parameters at initial diagnosis (pre). OR odds ratio, CI confidence interval

Odds ratios for specific long-term comorbidities were calculated in logistic regression analysis (Fig. 3). Hypertension at follow-up was significantly determined by older age (OR 1.078, 95% CI 1.028–1.132) and lower baseline UFC (OR 0.999, 95% CI 0.997–1.000). The odds of hyperlipidemia and obesity at last follow-up were significantly positively associated with higher baseline triglycerides (OR 1.007, 95% CI 1.001–1.013) and higher baseline BMI (OR 1.614, 95% CI 1.276–2.043), respectively. Presence of diabetes mellitus at last follow-up was determined by higher fasting glucose at diagnosis (OR 1.017, 95% CI 1.003–1.032); a negative association with UFC was borderline non-significant (OR 0.998, 95% CI 0.995–1.000).

## Discussion

Our study of 118 patients with Cushing’s syndrome in remission diagnosed and treated within a single tertiary center describes a large number of persisting comorbidities several years after remission of glucocorticoid excess, which can be independently predicted by age, fasting glucose, and UFC at diagnosis of Cushing’s syndrome. Lower UFC levels at diagnosis of Cushing’s syndrome are associated with a higher number of comorbidities not only in the whole cohort, but also in the pituitary and adrenal groups when analyzed separately. The most plausible basis for this association seems to be the fact that patients with lower baseline UFC exhibit a longer exposure time to excess glucocorticoids; and this relationship is much stronger in Cushing’s disease. The mild progression of clinical signs and symptoms in patients with lower baseline UFC might lead to a longer time until diagnosis. In addition, lower baseline UFC might be also linked to a smaller tumor size, which in turn makes successful localization and surgery difficult.

The prevalence of comorbidities in patients with Cushing’s syndrome in our cohort is higher than in the local general population [20, 21].

We find similar prevalences of comorbidities at Cushing’s diagnosis as shown in other publications [1, 6, 9]. Moreover, the total number of baseline comorbidities was not different between pituitary and adrenal cases, despite a higher degree of hypercortisolism in patients with Cushing’s disease (higher UFC values and higher cortisol after 1 mg dexamethasone test). The difference in the degree of hypercortisolism observed between pituitary and adrenal cases appears to depend on the study cohort, as other studies have described also lower and similar UFC levels in patients with Cushing’s disease when compared to adrenal Cushing’s syndrome [7, 10].

Remarkable is the nearly twice higher prevalence of diabetes mellitus in patients with Cushing’s disease, which was also observed by Giordano et al. [7]. While glucocorticoids impair both insulin secretion and insulin sensitivity, development of diabetes is primarily characterized by a beta-cell defect, which becomes more obvious in older patients with a positive family history [12]. In addition, diabetes mellitus was the only comorbidity previously found to correlate with the degree of hypercortisolism, highlighting the fact the amount of glucocorticoid excess plays a decisive role on beta-cell failure, on top of the already pre-existing environmental and genetic factors [8, 22]. The fact that remission of diabetes following the cure of Cushing’s syndrome was significantly higher in patients with Cushing’s disease and higher baseline UFC values supports the notion that the degree of hypercortisolism constitutes an additional and reversible stressor for beta-cell failure in these patients. On the other side, patients with adrenal disease and biochemically milder hypercortisolism experienced a significantly lower diabetes remission rate, highlighting genetic and environmental factors as major players in the context of milder Cushing’s syndrome. In a prospective study of metabolic and cardiovascular outcomes one year after remission of hypercortisolism, the adrenal cohort had slightly higher baseline mean UFC levels and a lower prevalence of metabolic and cardiovascular comorbidities at follow-up than the pituitary cohort [7]. Taken together, our data and the findings of Giordano et al. support the concept that long-term comorbidities after remission of hypercortisolism mainly depend on the baseline UFC levels at Cushing’s diagnosis, and not on the origin of hypercortisolism. This is also supported by our observation that the negative relationship between baseline UFC and long-term comorbidities remains significant also within the pituitary (*n* = 52) and adrenal (*n* = 58) groups.

In line with previously published data, hypertension is the most prevalent comorbidity at diagnosis of Cushing’s syndrome and resolves in 32.8% of cases after disease remission. Interestingly, we find higher baseline blood pressure values in patients with adrenal disease despite lower UFC, confirming previous findings [23]; a contributing factor to this difference in our cohort may be the higher age at diagnosis of patients with adrenal vs. pituitary disease (46.4 vs. 38.7 years). The prevalence of hypertension does not relate to the degree of hypercortisolism, but appears strongly dependent on the duration of glucocorticoid excess [8, 23]. These data might corroborate that higher blood pressure values in patients with adrenal Cushing’s syndrome and milder glucocorticoid excess might reflect longer disease duration, or longer exposure to hypercortisolism before disease diagnosis. In addition to the effects of glucocorticoids on vascular remodeling, hypertension per se might induce irreversible pathophysiological/vascular changes. Colao et al. [15] demonstrated a high degree of atherosclerosis 5 years after remission from Cushing’s disease. The authors discuss that the persistence of overweight/obesity and insulin resistance are the main reasons for the increased degree of atherosclerosis in these patients. Here, we show that older age and lower baseline UFC significantly predict the persistence of hypertension several years after remission of Cushing’s syndrome.

In the present cohort, overweight and obesity are still prevalent at follow-up. These data are in line with previous publications showing that most patients remain in the overweight or obese category also after remission from Cushing’s disease and despite a noticeable decrease in nearly all fat depots and improvement of fat distribution [24, 25]. Ten years after remission from Cushing’s syndrome, patients still display persistent accumulation of central fat and an unfavorable adipokine profile, both contributing to a state of persistent low-grade inflammation and to an increased degree of atherosclerosis [26]. So the cardiovascular risk profile improves after Cushing’s remission, but does not normalize [15]. BMI at diagnosis is the only factor predicting long-term obesity, and triglycerides at baseline are the only factor predicting hyperlipidemia at follow-up. In addition, weight increase long-term after remission of hypercortisolism was observed in patients with lower baseline UFC. The most likely explanation for this is the contributing effect of the increased long-term insulin resistance and subclinical inflammation in the development of obesity in patients with lower baseline UFC, which correlates with a higher long-term prevalence of prediabetes/diabetes.

Among all comorbidities, the highest remission rates were found for depression, confirming previous publications [6]. Nevertheless, patients with Cushing’s syndrome in remission experience more long-term psychopathology and maladaptive personality traits [27]. This is pathophysiologically linked to long-term effects of hypercortisolism on brain structure and functions, which seem to persist also several years after remission [28]. These disturbances may, at least in part, contribute also to the impaired quality of life several years after remission of glucocorticoid excess [29]. Depression at initial diagnosis and female gender are the main determinants of residual impairment of quality of life after Cushing’s remission [30, 31].

The most remarkable novel finding presented here is the predictive value of lower baseline UFC levels for the persistence of a higher number of long-term comorbidities. Specifically, lower UFC relates to the persistence of hypertension and diabetes mellitus. Hypertension is strongly affected by disease duration [8] and the most likely explanation for our finding is the longer time of exposure to excess glucocorticoids in patients with milder glucocorticoid excess.

Confirming all previous publications, we do not find any relationship between the degree of hypercortisolism and comorbidities at initial diagnosis, corroborating that not only the amount of glucocorticoid excess, but also the time of exposure to hypercortisolism or differences in glucocorticoid receptor sensitivity determine the phenotype of Cushing’s syndrome. In addition, we find that the number of comorbidities at baseline is per se a significant predictor of long-term comorbidities after cure. The present findings taken together with results from previous publications support the hypothesis that the severity and consequences of Cushing’s syndrome might be better assessed by evaluating the clinical presentation and associated comorbidities, than by the magnitude of cortisol excess. The persistence of comorbidities and cardiovascular risk factors leads to an increased mortality, also dozens of year after disease remission [4].

The strength of the present study is the accurate characterization of patients in one tertiary center with a single laboratory and standard protocols for the diagnosis and follow-up of patients with Cushing’s syndrome, including screening and treatment of Cushing’s comorbidities. The inclusion of patients from a limited geographical area is also a limitation. In addition, the study was not designed for testing baseline differences between adrenal and pituitary Cushing’s and disease cure/mortality rates, but describes factors predicting comorbidities in patients with Cushing’s syndrome in remission. To date, there is no evidence for a possible relationship between baseline UFC and remission/death rates in Cushing’s disease, but mortality strongly depends on the exposure time to excess glucocorticoids, calculated from the duration of preoperative symptoms until the achievement of postoperative remission [3].

In summary, the high prevalence of long-term comorbidities after remission of Cushing’s syndrome underpins the importance of long-term monitoring and disease-specific therapy for reducing cardiovascular mortality. Factors predicting long-term comorbidities are older age and hyperglycemia at diagnosis, as well as lower baseline 24-h urinary cortisol excretion, reflecting longer exposure time to excess glucocorticoids. This finding highlights the importance of early diagnosis, but also the necessity to immediately initiate treatment also in mild cases of Cushing’s syndrome.

## Supplementary information

Supplemental Table 1
