# Supplementary material for: Factors predicting long-term comorbidities in patients with Cushing’s syndrome in remission
Source: Endocrine. 2018 Nov 22;64(1):157–68. doi: 10.1007/s12020-018-1819-6 (PMC6453862; doi:10.1007/s12020-018-1819-6)
Supplement: Supplementary file 1 — Supplemental Table 1 [file 12020_2018_1819_MOESM1_ESM.docx]

**Supplemental table 1.** Remission rates and rates of new manifestation of comorbidities in the whole cohort as well as in patients with Cushing’s diseases and adrenal Cushing’s syndrome.

|  | **Remission** | | | |
| --- | --- | --- | --- | --- |
|  | **All patients** | **Pituitary** | **Adrenal** | **Ectopic** |
| **Hypertension** | 36.4% | 31.9% | 34.6% | 75.0% |
| **Hyperlipidemia** | 22.6% | 26.8% | 17.4% | 33.3% |
| **Overweight** | 44.2% | 41.2% | 52.2% | 0.0% |
| **Obesity** | 44.1% | 30.8% | 56.3% | 40.0% |
| **Prediabetes** | 83.3% | 100% | 71.4% | 100.0% |
| **Diabetes mellitus** | 56.3% | 63.2% | 25.0% | 80.0% |
| **Metabolic syndrome** | 60.0% | 66.7% | 40.0% | 85.7% |
| **Depression** | 51.6% | 60.0% | 35.3% | 100.0% |
| **Osteoporosis** | 17.9% | 26.7% | 10.0% | 0.0% |
|  | **New manifestation** | | | |
|  | **All patients** | **Pituitary** | **Adrenal** | **Ectopic** |
| **Hypertension** | 12.5% | 0% | 25.0% | - |
| **Hyperlipidemia** | 40.9% | 40.0% | 40.0% | 50.0% |
| **Overweight** | 11.4% | 9.1% | 15.6% | 0.0% |
| **Obesity** | 3.8% | 5.4% | 2.6% | 0.0% |
| **Prediabetes** | 1.3% | 0% | 2.9% | 0.0% |
| **Diabetes mellitus** | 1.2% | 0% | 2.1% | 0.0% |
| **Metabolic syndrome** | 7.9% | 7.4% | 8.6% | 0.0% |
| **Depression** | 9.8% | 15.4% | 5.1% | 0.0% |
| **Osteoporosis** | 10.3% | 7.4% | 10.8% | 25.0% |

Data are shown as percentages of patients with comorbidities at diagnosis of Cushing’s syndrome, who had a remission until the last follow-up visit (Remission) and percentages of patients with no comorbidity at baseline and new disease manifestation until the last follow-up visit (New manifestation).
